# Water Kefir Grains—Microbial Biomass Source for Carbonaceous Materials Used as Sulfur-Host Cathode in Li-S Batteries

**DOI:** 10.3390/ma15248856

**Published:** 2022-12-12

**Authors:** Ana L. Páez Jerez, M. Fernanda Mori, Victoria Flexer, Alvaro Y. Tesio

**Affiliations:** 1 CIDMEJu (CONICET-Universidad Nacional de Jujuy), Centro de Desarrollo Tecnológico General Savio, Av. Martijena S/N, Palpalá 4612, Argentina; 2 Instituto de Investigaciones en Catálisis y Petroquímica, CONICET, Facultad de Ingeniería Química, Universidad Nacional del Litoral, Santiago del Estero 2829, Santa Fe S3000, Argentina

**Keywords:** biomass-derived carbon, non-activated carbon, water kefir grains, cathode material, lithium–sulfur battery

## Abstract

Nowadays, the use of biomass to produce cathode materials for lithium–sulfur (Li-S) batteries is an excellent alternative due to its numerous advantages. Generally, biomass-derived materials are abundant, and their production processes are environmentally friendly, inexpensive, safe, and easily scalable. Herein, a novel biomass-derived material was used as the cathode material in Li-S batteries. The synthesis of the new carbonaceous materials by simple carbonization and washing of water kefir grains, i.e., a mixed culture of micro-organisms, is reported. The carbonaceous materials were characterized morphologically, texturally and chemically by using scanning electron microscopy, N_2_ adsorption–desorption, thermogravimetric analysis, X-ray diffraction, and both Raman and X-ray photoelectron spectroscopy. After sulfur infiltration using the melt diffusion method, a high sulfur content of ~70% was achieved. Results demonstrated that the cell fitted with a cathode prepared following a washing step with distilled water after carbonization of the water kefir grains only, i.e., not subjected to any chemical activation, achieved good electrochemical performance at 0.1 C. The cell reached capacity values of 1019 and 500 mAh g^−1^ sulfur for the first cycle and after 200 cycles, respectively, at a high mass loading of 2.5 mg_S_ cm^−2^. Finally, a mass loading study was carried out.

## 1. Introduction

Energy demand plays an important role in modern society. The increasing consumption and depletion of non-renewable fossil fuels (i.e., oil, coal and natural gas) as well as the environmental risks generated by CO_2_ emissions constitute a strong incentive to produce other electrical energy systems derived from natural and environmentally friendly sources [1]. The gradual replacement of internal combustion engine vehicles with cleaner means of transportation, such as electric vehicles, is another crucial step for our society [2,3]. Among the alternative energy sources, wind and solar stand out. However, up to now these applications still do not compete with devices powered by fossil fuels [4]. Besides, due to the intermittent nature of these energy sources, it is imperative to use energy storage systems.

At the present time, no alternative rechargeable battery technology has shown a stability and long cycling capability that can be compared to Li-ion batteries. Thus, the search for devices that can achieve higher energy density and specific capacity continues to be a research focus and a great challenge [5,6]. Among the new generation of electrochemical energy storage devices, lithium–sulfur (Li-S) batteries constitute a promising alternative, demonstrating the potential to offer huge economic benefits and improve lifestyles globally [4,7,8,9]. Sulfur is theoretically able to deliver a high capacity and large energy density of 1675 mAh g^−1^ and 2600 Wh kg^−1^, respectively, with natural abundance and low cost. Unfortunately, the practical discharge capacity and cycling of Li-S batteries are affected by several problems due to the complex multi-electron redox processes, occurring at both the anode and cathode. At the anode, a common problem is related to uncontrolled dendritic Li growth, while at the cathode side, the most serious problems are due to the insulating nature of both elemental sulfur and the final discharging products, Li_2_S_2_/Li_2_S and the shuttle effect caused by the soluble polysulfides in the electrolyte [10].

With the objective of tackling the problems at the cathode, it becomes imperative to add conductive matrices to transfer electrons, while serving as sulfur hosts. Many different materials have been combined with sulfur, and these can be generally classified into two categories. The first one includes non-carbonaceous materials such as metallic oxides, metallic sulfides, and conductive polymers which have the ability to better accommodate the volume changes and can establish strong bonds with sulfur [11,12,13,14]. The second group is formed by a very large variety of carbonaceous materials, which are ideal coating materials due to their high conductivity, great abundance, dense structure and elastic nature, and can be obtained alternatively from commercially available carbon sources or synthesized from sustainable resources through relatively simple procedures [13,15,16,17].

A particularly interesting sustainable alternative for cathodes in Li-S batteries is biomass-derived material. The term biomass refers to living matter (e.g., plants, animals and micro-organisms) in the environment [18]. The first article on the topic dates back to 2011 [19] and from that moment the number of publications in this field has increased remarkably. Although most carbons derived from biomass come from organic waste, there are very few reports about the use of carbons derived from micro-organisms in energy storage and production, especially in Li-S battery applications [20]. A clear note should be made, that we are not referring to actually using the living cells as biocatalysts, we are only referring to carbonized materials. Xia et al. reported an activation method based on biological fermentation, a facile and eco-friendly approach, to prepare a carbon precursor derived from yeast-fermented banana peels used as a cathode material in Li-S batteries with a sulfur content of 74.34%. The cell achieved an initial capacity of 1174 mAh g^−1^ sulfur at 0.1 C and capacity retention of 58.35% after 100 cycles [21]. Although in this work the authors used banana peel waste to prepare a porous carbon, banana peels were combined with yeasts, which serve as an activating agent, avoiding in this way the use of chemical activators. Wu et al. reported a micro-organism-route to synthesize fungus that could be used as a sulfur host material for lithium–sulfur batteries. As a result, the cell reached an initial capacity of 1319 mAh g^−1^ sulfur and 663 mAh g^−1^ sulfur after 100 cycles at 0.1 C [22]. Zhao et al. used green algae to prepare a host material to encapsulate sulfur for Li-S batteries with a sulfur content of 63%. When tested at 0.1 C, the cell yielded capacity values of 1327 and 757 mAh g^−1^ sulfur for the first cycle and over 100 cycles, respectively [23].

Most of the articles in the literature discussed carbon compounds produced from bio-mass that were activated chemically or physically to achieve desirable characteristics and good electrochemical performances. Cathode materials made of non-activated carbons are unusual; some examples are carbons made from filter papers [24], wood fibers [25], rice husks [26], and plane tree bark [27]. Li-S batteries assembled with these cathodes achieved good capacity values and long-term cycling, and no chemicals or activation techniques were needed, making it an economical and environmentally beneficial method of generating carbons that makes use of the natural components of these waste materials.

Water kefir also known as *sugar(y) kefir*, *Tibicos*, *Tibi*, *Tibetan mushroom*, or *kefir d’ aqua* is a sparkling and slightly acidic fermented beverage inoculated with water kefir grains. These grains of jelly-like consistency are a symbiotic culture harbored by an exopolysaccharide, where lactic acid bacteria, yeast and acetic acid bacteria are the main microbial community [28]. They putatively have potential benefits and/or uses as dietary supplements, or probiotics, reducing blood cholesterol and balancing the intestinal microbiota [29]. In addition, they have been reported to serve in the development of biodegradable films [30], and as sources of biopolymers to form new materials with different applications [31].

**Figure 1:**
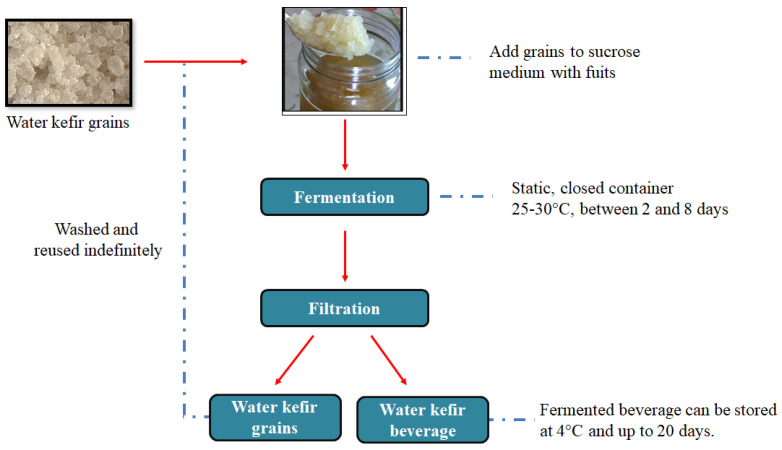
Traditional production of water kefir.

In the present work, we report a new material synthesized from microbial biomass, water kefir grains, which were used as cathode in Li-S batteries. They are a symbiotic culture of micro-organisms and their production is straightforward and traditionally produced on a home-scale without major inconvenience, resulting in a beverage which is used for human consumption (see Figure 1) [28,32]. Therefore, the carbonaceous material was obtained by simple carbonization of dried water kefir grains at 800 °C for 1 h under nitrogen atmosphere, without being subjected to an activation process (neither chemical, nor physical). Two types of water kefir carbons were obtained: unwashed and washed with distilled water only. These two samples were mixed with sulfur using melt diffusion method in order to obtain composites with high sulfur content of ~70%. The water kefir carbons were first characterized texturally, morphologically and chemically using different techniques, and then tested as cathodes in Li-S cells. Both cells exhibited good electrochemical performance at 0.1 C (1 C = 1675 mAh g^−1^) in ether-based electrolyte with LiNO_3_ as additive. The cell prepared with water kefir carbons, which were only washed, achieved an initial capacity of 1019 mAh g^−1^ sulfur at a high mass loading of 2.5 mg_S_ cm^−2^. Relatively high-capacity retention values were recorded, with values larger than 600 and ~500 mAh g^−1^ sulfur recorded after 100 and 200 cycles, respectively.

## 2. Materials and Methods

### 2.1. Preparation of Carbonaceous Material

Water kefir grains were subjected to a drying pre-treatment at 110 °C overnight. The dried material was carbonized from room temperature to 800 °C for 60 min with a heating ramp of 15 °C min^−1^ in a tubular furnace (Indef, T-150, Córdoba, Argentina) under a nitrogen atmosphere. The yield of water kefir carbonization was 15%. After that, the water kefir carbons were milled at 300 RPM during 60 min by using a planetary ball mill (PM 100, RETSCH, Haan, Germany). After mechanical treatment, the product can be washed, or not, in order to prepare the composite cathode. These were termed WKC-NW and WKC-W, referring to unwashed and washed water kefir carbons.

### 2.2. Preparation of Carbon/Sulfur Composite

To prepare the composites, sulfur (powder, >99%, Biopack, Sydney, Australia) was added to the water kefir carbons described above, using the melt diffusion method. Generally, the materials were mixed inside a glove box (MBRAUN Unilab Pro SP, with H_2_O and O_2_ contents bellow 1 ppm and 0.1 ppm) in a S:water kefir carbon ratio of 70:30 (*w*/*w*), and then taken out in a sealed round-bottom flask. The sealed flask was heated at 155 °C in a glycerine bath for 4 h. In this process, the temperature is adjusted to just above the melting point of sulfur, where the viscosity is lowest and sulfur powder melts, diffuses and reacts, dispersing uniformly in the porous carbon structure [33,34,35]. The final composites were named WKC-NW/S and WKC-W/S, and the sulfur content, determined by thermogravimetric analysis was 70 and 68%, respectively.

### 2.3. Water Kefir Carbon and Composite Material Characterization

Scanning electron microscopy (SEM, Carl Zeiss, Jena, Germany, EVO MA10 Model, equipped with a LaB_6_ filament), operating at 15 kV, with a focus between 6 and 7 mm, in a variable pressure mode in the chamber and using a secondary electron detector, was employed to study the morphology of water kefir carbons. N_2_ adsorption–desorption isotherms at 77 K were measured using a Micromeritics-ASAP 2000 instrument. Previously, samples were degassed at 200 °C for 12 h. The specific surface areas (S_BET_) were derived from N_2_ adsorption–desorption isotherms by means of the BET (Brunauer, Emmet and Teller) equation. The pore size distributions and medium pore diameter were determined using the Density Functional Theory (DFT). The micropore volumes (V_µP_) were obtained by α-plot model and the total volumes of porous (V_TP_) by Gurvich rule at *p*/*p*_0_ = 0.95. Thermogravimetric analysis (TGA) was performed in a thermogravimetric analyzer (TGA-50, SHIMADZU, Kyoto, Japan) in a temperature range from room temperature to 800 °C under an O_2_ atmosphere and at a heating rate of 10 °C min^−1^ in order to assess the carbon content; and in a temperature range from room temperature to 500 °C under an N_2_ atmosphere, at a heating rate of 5 °C min^−1^, in order to determine the sulfur content of the samples. X-ray diffraction (XRD) patterns were recorded with a PHILIPS PW1710 X-ray diffractometer with a copper anode and curved graphite monochromator, operating at 45 KV and 30 mA. The scanning conditions for structural analysis were recorded over a 2θ range from 10° to 80°. Raman spectroscopy was performed on a WITEC UHTS 300 spectrometer using 532 nm laser irradiation. X-ray photoelectron spectroscopy (XPS) was carried out in a Specs multi-technique spectrometer equipped with a dual X-ray source (Ag/Al) and a hemispherical analyzer (PHOIBOS 150) in fixed analyzer transmission (FAT) mode. The spectra were obtained with a step energy of 30 eV with monochromatic radiation Al Kα at 300 W. The pressure during measurements was less than 10^−9^ mbar. Spectra were fitted in order to identify the different functional groups in the materials. Before measurements, the samples were firstly heated at 250 °C for 1 h. Then, they were subjected to evacuation under ultra-high vacuum for at least 12 h. The analysis of the spectra was carried out using the software CASAxps. The peak positions were corrected by the surface charge, using the C 1s at 285 eV, typical of the contamination of the samples. Baseline correction was performed with a Shirley function. The assignment of the peaks was carried out by comparison with data bases and similar works. In the deconvolution of the peaks, 30% Lorentzian–Gaussian functions were used. The width at mid-height of the peaks (fhwm) was always set at 2 eV [36].

### 2.4. Cathode Preparation

The electrodes were prepared by mixing the synthetized material (WKC-NW/S and WKC-W/S), PVDF (powder, GELON LIB GROUP) and carbon Super P (GELON LIB GROUP, type C45) in a mass ratio of 85:10:5. All components were dispersed in N-methyl-2-pyrrolidene (NMP, >99.5%, Merck, Darmstadt, Germany). The resultant slurry was obtained by stirring for at least 12 h and then uniformly spread onto a gas diffusion layer (GDL) carbon cloth with a microporous layer (GELON LIB GROUP, thickness 0.41 mm) using the doctor-blade technique at different heights. The final sulfur content in these deposits was 59.5 and 57.8% S for WKC-NW/S and WKC-W/S, respectively. Finally, it was dried in a vacuum oven (ARCANO DZF60-20) at 50 °C for 12 h. The areal loading was in the range of 0.3–2.5 mg_S_ cm^−2^ and the cathode areal loadings were between 0.4 and 3.6 mg cm^−2^.

### 2.5. Electrochemical Characterization

Coin-type cells (CR2032) were assembled in a glove box (MBRAUN Unilab Pro SP) with H_2_O and O_2_ contents below 1 ppm and 0.1 ppm, respectively, using a lithium metal disc as anode (diameter of 15.6 mm and thickness of 0.45 mm), and polypropylene as separator (Celgard 2400, diameter of 16 mm and thickness of 25 μm), all components from GELON LIB GROUP. The electrolyte used (30 µL) was 0.8 M lithium bis (trifluoromethanesulfonyl) imide (LiTFSI, >99%, Sigma-Aldrich, St. Louis, MO, USA) and 0.3 M LiNO_3_ (Sigma-Aldrich) dissolved in a solvent mixture of 1,3-dioxolane (DOL, GELON LIB GROUP) and 1,2-dimethoxyethane (DME, GELON LIB GROUP) with a volume ratio of 1:1. The electrolyte-to-sulfur (E/S) ratio was 35–60 µL mg^−1^. Galvanostatic measurements were carried out in an 8-channel battery tester analyzer (Neware). The cells were discharged and charged galvanostatically (GDC) at 0.1 C (1 C = 1675 mAh g^−1^) for their long-term cycling performance and at various cycling rates from 0.1 to 1 C for their rate capability study, and between 1.7 and 2.7 V, at room temperature. Specific capacities and current densities were calculated based on the sulfur content determined by TGA. Cyclic voltammetries (CV) were measured in a VMP3 BioLogic multichannel galvanostat–potentiostat with a potential range from OCP (open circuit potential) to 1.7 V at different scan rates of 0.005, 0.010, 0.015 and 0.020 mV s^−1^. Electrochemical impedance spectroscopy (EIS) was also performed in a VMP3 BioLogic multichannel galvanostat-potentiostat from 1 MHz to 10 mHz with a voltage amplitude of 5 mV.

## 3. Results and Discussion

### 3.1. Structural, Morphological and Chemical Properties

**Figure 2:**
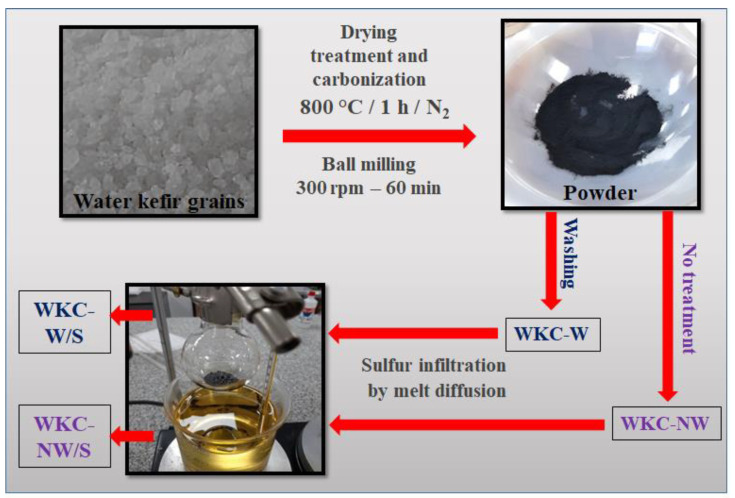
Scheme of the synthesis procedure for the WKC-NC/S and WKC-W/S composites.

To the best of our knowledge, no articles about this kind of microbial biomass-derived carbons used as a cathode material in Li-S batteries have been reported. The different synthesis steps for preparation of the two kinds of water kefir carbons with the aim to be used as sulfur hosts are summarized in Figure 2. The carbonization step at 800 °C for 1 h in an N_2_ gas atmosphere was used to cleave the C, H and O bonds, and eliminate volatile compounds and other gas spillovers, thus preparing a carbonaceous matrix with a large variety of pore sizes. It has been reported that the usual pyrolysis method to prepare biomass-derived carbons can be divided into three different stages: below 400 °C, 400–700 °C and 700–1000 °C [37]. In the first stage, the raw material will mainly undergo a decomposition reaction (i.e., deacidification and dehydration), but the C-O bonds do not decompose. However, in the second stage, the bonds containing O will break and they will be eliminated in the form of CO and CO_2_. Besides, volatile compounds derived from the raw material are gradually decreased. Finally, when the carbonization temperature is in the range of 700–1000 °C, a dehydrogenation reaction is carried out, obtaining a carbonaceous matrix with pores of different shapes and sizes.

**Figure 3:**
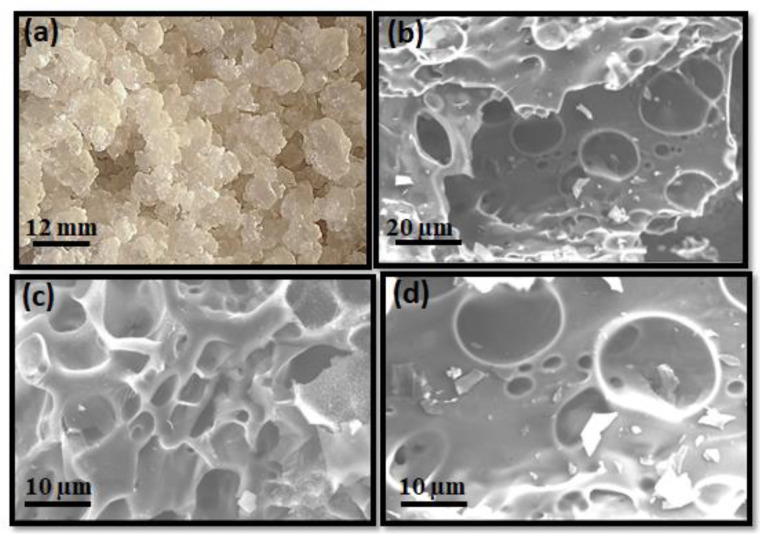
Optical image of water kefir grains (**a**) and SEM images obtained for unwashed water kefir carbons measured at 20 (**b**) and 10 µm (**c**,**d**).

The morphologies and structures of water kefir samples, after being carbonized and milled, are shown in Figure 3. Water kefir grains are gelatinous structures of 5 to 20 mm diameter and with an irregular, cauliflower-like shape. The SEM images of water kefir carbons depict a three-dimensional structure of grainy appearance with interconnected and randomly oriented pores, closed by compact walls in an uneven surface. No significant differences were observed between the WKC-NW and WKC-W samples. The morphology of washed water kefir carbons can be seen in Figure S1.

**Figure 4:**
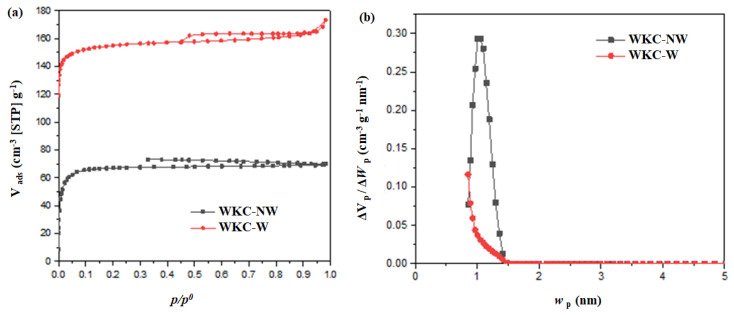
Nitrogen adsorption–desorption isotherms at 77 K for WKC-NW and WKC-W (**a**) and their micropore size distribution (**b**).

The N_2_ adsorption–desorption isotherms and pore size distribution profiles of WKC-NW and WKC-W derived from BET measurements are depicted in Figure 4. For the studied pressure range, we can see that both samples show high and stable adsorption, although it is higher for WKC-W. At low relative pressure, the N_2_ adsorption–desorption isotherms can be regarded as type I based on IUPAC classification, indicating the presence of micropores. Besides, it can be noticed that both desorption curves are incomplete. This could be a consequence of the presence of surface groups or very narrow micropores where the gas molecules are trapped. The resulting porosity data including BET specific surface area (S_BET_), total pore volume (V_TP_) and micropore volume (V_µP_) for WKC-NW and WKC-W are summarized in Table 1. Compared with WKC-NW, WKC-W developed a specific surface area 2.3 times larger, twice the value of total pore volume, and a micropore volume that represents 85% of microporosity. A higher S_BET_ could indicate more defects in the WKC-W structure and more space to store sulfur and restrict more polysulfides. In short, WKC-W exhibited a larger S_BET_ with smaller pores, but larger pore volumes compared to WKC-NW. This increase in S_BET_ may be the result of the elimination during washing of chemicals that offer the sample weight but no area and may be clogging the pores, rendering them inaccessible [38].

**Figure 5:**
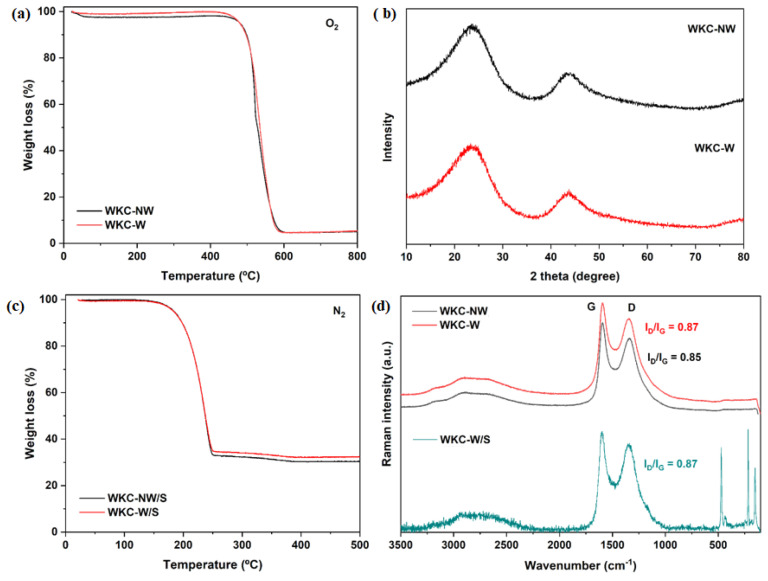
TG curves in oxygen (**a**), XRD patterns (**b**), TG curves in nitrogen (**c**) and Raman spectra (**d**) for WKC-NW, WKC-W and WKC-W/S.

The TG curves, XRD patterns and Raman spectra for water kefir carbons, washed and unwashed, and TG curves after sulfur infiltration and Raman spectrum of WKC-W/S, are displayed in Figure 5. The TG analyses were first performed under oxygen atmosphere in order to determine the carbon content of the samples. The results confirmed materials with high carbon content, 95% for both WKC-NW and WKC-W. A high carbon content is a necessary condition for suitable biomass-derived carbons [39]. Moreover, it means that the ash content is low, which is in agreement with data for other biomass-derived chars and commercial carbons reported by Ahmedna et al. [40], and consequently, the non-electroactive impurity content is also low. XRD patterns were very similar for the two studied samples. The diffractograms depict two broad peaks of low intensity centered at 25° and 43°, respectively. These two peaks correspond to the crystallographic planes of graphitic carbon (0 0 2) and (1 0 0), respectively [41]. The widths of the signals indicates a low crystallinity corresponding to carbons with a disordered structure [35,42].

After sulfur infiltration, the sulfur contents of the composites were determined by TGA under N_2_ atmosphere (see Figure 5c). The results confirmed sulfur contents of 70 and 68% for WKC-NW/S and WKC-W/S, respectively. These values can be considered very close to each other, considering the intrinsic error in thermogravimetric analysis. The structure of WKC-NW and WKC-W (before and after sulfur infiltration) was analyzed by Raman spectroscopy in the range of 3500–100 cm^−1^. The Raman spectra showed a typical profile of poorly graphitized carbon, which is consistent with the XRD pattern. The well-known G and D bands, that indicate graphitic carbon and defects in the crystalline graphite structure, were observed at 1598/1337 cm^−1^ and 1593/1346 cm^−1^ for WKC-NW and WKC-W, respectively. Besides, since the D band is ascribed to a structural disorder and grows in intensity as the disorder increases, the relative intensity of these two bands is indicative of the degree of order in the carbon materials [35]. The *I_D_*/*I_G_* values were 0.85 and 0.87 for WKC-NW and WKC-W, respectively, indicating no major changes with washing. After impregnation with sulfur, the bands corresponding to the stretching and bending modes of elemental sulfur can be clearly seen, appearing at 475, 224 and 157 cm^−1^ [43], respectively, but they do not affect the G and D carbon bands, observed at 1598 and 1346 cm^−1^, respectively. Besides, the *I_D_*/*I_G_* value was not modified after sulfur infiltration, implying that no additional lattice defects emerged.

**Figure 6:**
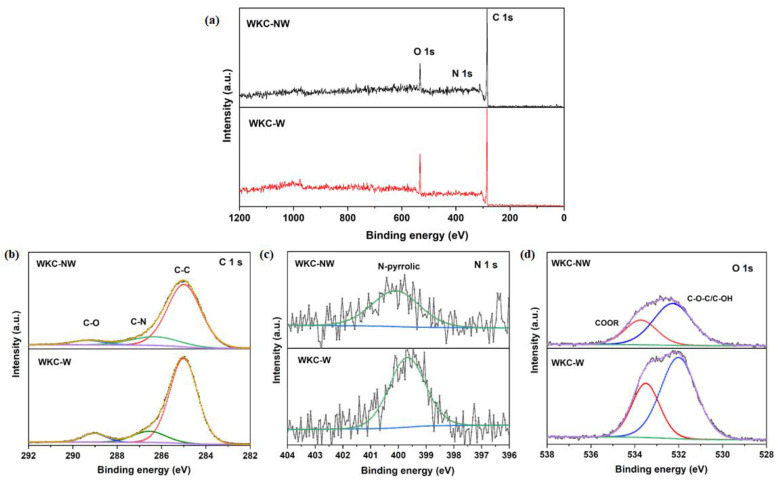
Survey spectra (**a**), high resolution C 1 s (**b**), N 1 s (**c**) and O 1 s (**d**) XPS spectra obtained for WKC-NW and WKC-W.

The full XPS spectra indicated surface elemental compositions for C, O and N of 83.6%, 15.4% and 0.9% for WKC-W, while the elemental contents determined for WKC-NW were 89.0%, 9.9% and 1.1%, respectively, indicating that both water kefir carbons are doped with O and N (see Figure 6). The oxygen peak was very high in both spectra, indicating that the large O content present in the raw material is not removed during the pyrolysis process. Besides, it could be seen that the oxygen content increased with washing, decreasing the carbon content. The high resolution C 1 s spectrum for WKC-NW can be divided into three bands at 284.9, 286.2 and 289.3 eV regions, corresponding to C-C, C-N and C=O bonds, respectively. The corresponding bands for WKC-W were observed at 285.0, 286.5 and 289.0 eV [44,45]. The high resolution N 1 s indicated the presence of a single species at 400.0 and 399.7 eV for WKC-NW and WKC-W, respectively, which can be ascribed to N-pyrrolic in agreement with the literature [44,45,46]. The deconvolution of O 1 s spectrum for WKC-NW revealed two main components at 532.5 and 533.9 eV, ascribed to C-O-C/C-OH and COOR, respectively. The same components for WKC-W were centered at 532.0 and 533.49 eV [44,45]. The peak area for the component at lower eV in the high resolution O 1 s was compared with the area of the more intense component of C 1 s and they were 0.16 and 0.42 for WKC-NW and WKC-W, respectively, indicating once again the increase in hydroxyl groups. Besides this, no major differences were found in the surfaces of both carbonaceous materials as a result of the washing process.

### 3.2. Electrochemical Properties

**Figure 7:**
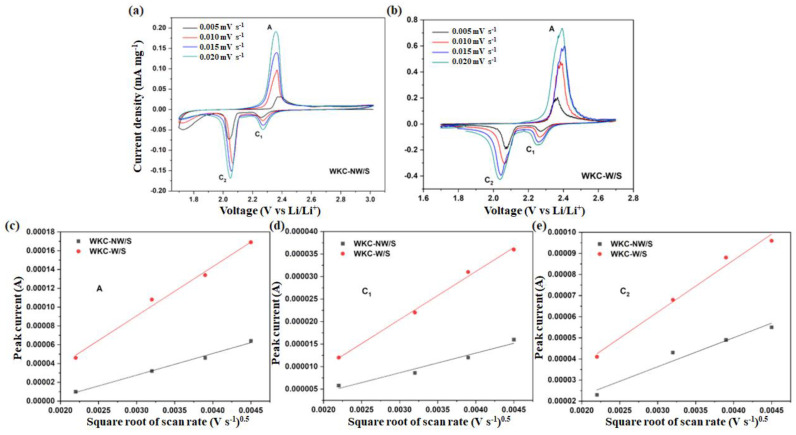
CV profiles for WKC-NW/S and WKC-W/S at various scan rate (**a**,**b**) and plots of the CV peak current of the A, C_1_ and C_2_ peaks for both electrodes vs square root of scan rate (**c**–**e**).

In order to evaluate the electrochemical properties of the water kefir carbons, the cells with WKC-NW/S and WKC-W/S were first tested by cyclic voltammetry (CV) in order to assess the lithium ions’ diffusion properties. The profiles are shown in Figure 7. Figure S2 shows three consecutive CVs where the signals are reproducible, proving the stability of the system. The CV curves obtained for both composites depict a typical redox mechanism of sulfur-containing carbon electrodes with two reduction peaks, C_1_ and C_2_, and a simple anodic peak A. In the cathodic scan, the reduction peak C_1_ is attributed to the conversion of elemental sulfur to long chain polysulfides (Li_2_S_n_, 4 ≤ n ≤ 8). The second reduction peak C_2_ is corresponded to the reduction of high-order lithium polysulfides to Li_2_S_2_ and Li_2_S. In the anodic scan, the peak A is due to the reversible conversion of Li_2_S_2_/Li_2_S to lithium polysulfides/elemental sulfur [35,47,48]. A careful examination of the reduction peaks for WKC-NW/S, shows a reduction peak at voltage values below 1.80 V, suggesting the presence of some electroactive species which does not interfere with the species of interest to battery performance. That peak disappeared on the washed carbon, as can be seen from the CV curves of WKC-W/S.

In addition to adsorption, the rapid charge transfer is another important factor that accelerates the conversion of polysulfides. As shown in Figure 7c–e, there is a linear correlation between peak currents and the square root of the scan rate, and thus the Li^+^ diffusion coefficient (D) can be determined from the Randles–Sevcik equation:

I_p_ = (2.69 × 10^5^) n^1.5^SD^0.5^Cν^0.5^ (1)

where I_p_ is the peak current, n is the number of electrons transferred, S is the active electrode area, C is the Li ion concentration and *ν* is the scan rate [49,50,51,52]. The WKC-NW/S cathode yielded a lower slope, indicating that the diffusion of electroactive species, in all three redox peaks, is less effective. Diffusion of Li^+^ is known to be one of the main limiting processes in Li-S batteries, thus higher diffusion coefficients eventually correlate with better lithium polysulfide redox kinetics in the discharge/charge processes. The Li^+^ diffusion coefficients obtained for both discharge (C_1_ and C_2_) and charge (A) processes are summarized in Table 2. The Li^+^ diffusion coefficients are comparable to others reported in the literature [53,54,55].

**Figure 8:**
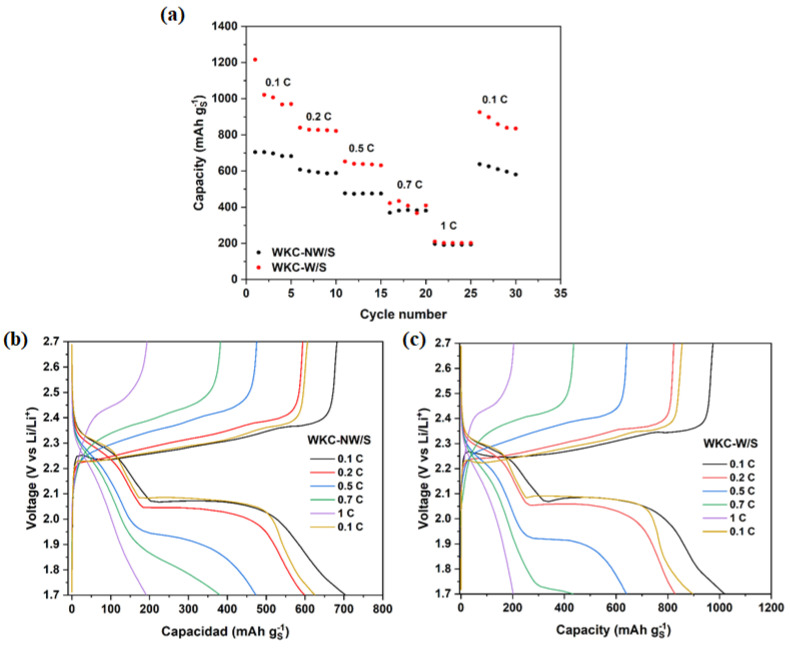
Rate capability analysis for WKC-NW/S and WKC-W/S (**a**), GDC profiles for the second cycle at different C-rates for WKC-NW/S (**b**) and WKC-W/S (**c**).

Figure 8 summarizes the rate capability experiments for WKC-NW/S and WKC-W/S increasing C-rate from 0.1, 0.2, 0.5, 0.7 to 1 C rates and returning it to 0.1 C. Both cells achieved good sulfur utilization and high reversible capacity values at the low and medium C-rate; being always better for washed material. The cathodes made with washed and unwashed water kefir carbons achieved 1021 and 704 mAh g^−1^ sulfur, respectively, for the second cycle. Both cells kept stable cycling for five cycles as the cycling rates increase to 1 C rate. The cells were able to recover a high percentage of their initial capacities when the C-rate value returned to 0.1 C (94% for WKC-NW/S and 95% for WKC-W/S), showing the robustness of the system.

**Figure 9:**
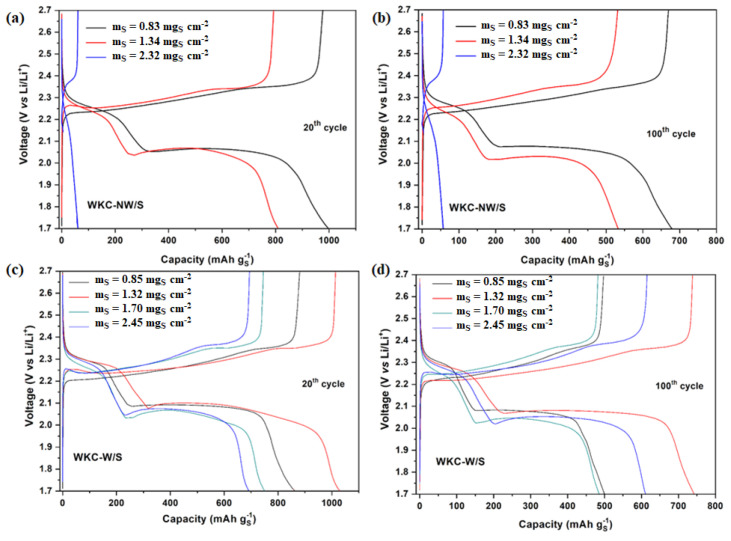
Discharge/charge profiles at 0.1 C for the 20th and 100th cycles at different mass loadings for WKC-NW/S (**a**,**b**) and WKC-W/S (**c**,**d**).

**Figure 10:**
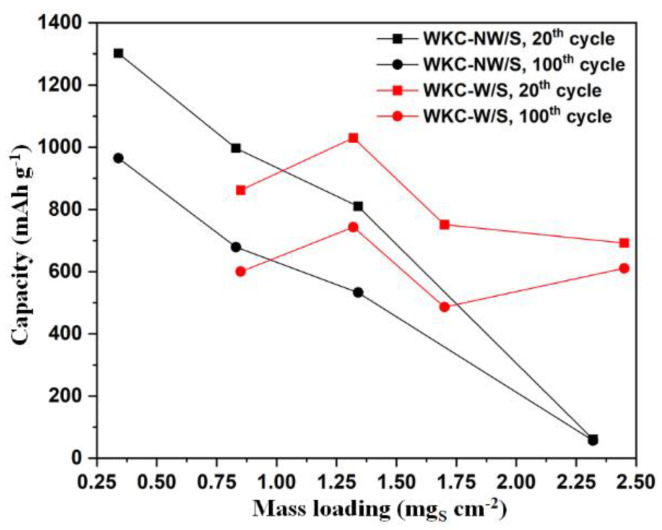
Comparison of the capacity values for the 20th and 100th cycles vs sulfur mass loading for WKC-NW/S (black curves) and WKC-W/S (red curves).

The galvanostatic discharge/charge profiles of WKC-NW/S and WKC-W/S cathodes at 0.1 C for the 20th and 100th cycles for different mass loading (0.8–2.5 mg_S_ cm^−2^) are shown in Figure 9 (Figure S3 shows the first three cycles at 0.1 C for both cells). The discharge curves show two voltage plateaus whereas a single voltage plateau is noticeable in the charge curves, which is consistent with the peaks observed in the CVs (Figure 7a,b). From the graphs, it can be seen that at low mass loadings, the cells exhibit the best electrochemical performances for the 20th and 100th cycles, being better at 0.8 mg_S_ cm^−2^ for WKC-NW/S and at 1.3 mg_S_ cm^−2^ for WKC-W/S. The experimental specific capacity values, at the high mass loading value of 2.45 mg_S_ cm^−2^ are of 692 and 611 mAh g^−1^ sulfur for the 20th and 100th cycles, respectively, in contrast WKC-NW/S only reaches capacity values of 61 and 57 mAh g^−1^ for the same cycles and at a similar mass loading. This trend can be clearly seen in Figure 10. Once again, the galvanostatic discharge/charge curves of the cells for the 20th and 100th cycles are very similar for each type of water kefir carbons, being the capacity fading practically linearly for the case of WKC-NW/S when the mass loading increases.

Moreover, upon careful inspection of the shape of the discharge curves, it can be noticed that the voltage drop is not abrupt from the inflection point towards the end of the discharge plateau for WKC-NW/S. This gradual drop is attributed to more complex and sluggish redox processes. In contrast, WKC-W/S showed faster and more complete reduction processes where the voltage falls suddenly at the same inflexion point. Another improvement that was achieved with the washing of the carbon was the reduction of the polarization of the cell, i.e., the voltage gap in the charge and second discharge plateaus (ΔΕ) calculated at 50% discharge capacity. Values of 240/220 mV vs 170/200 mV for the 20th and 100th cycles for WKC-NW/S and WKC-W/S, respectively, at a mass loading value of 0.8 mg_S_ cm^−2^ were recorded. Likewise, the corresponding ΔΕ values at a mass loading of 1.3 mg_S_ cm^−2^ were 230/290 mV and 180/200 mV for the 20th and 100th cycles for WKC-NW/S and WKC-W/S, respectively.

EIS experiments for WKC-NW/S and WKC-W/S were also performed before and after five cycles at 0.1 C (see Figure S4). The plots showed a semicircle in the middle to the high-frequency zone which is related to charge transfer resistance by the electrode reaction kinetics. Although it was not expected, the charge transfer resistance for WKC-NW/S was lower than the one for WKC-W/S. However, after 5 cycles at 0.1 C, both cycled cells reduced their charge transfer resistance. This lower resistance, added to a greater decrease in resistance after cycling in the case of the unwashed material, indicates that the key factors that determine the performance of the cell are pore area and pore volume which are higher in the case of the washed material. Therefore, it can be stated that the better conductivity of the unwashed material cannot be compensated by the increase in area obtained by washing the material.

**Figure 11:**
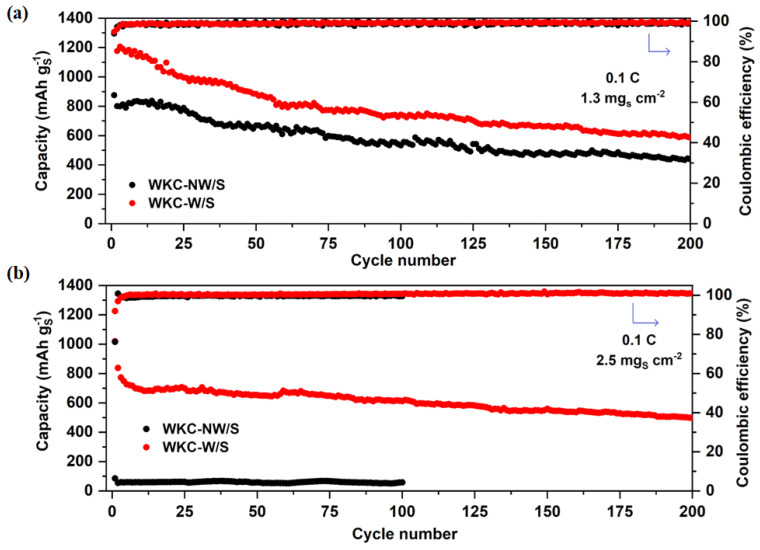
Long term cycling performances at 0.1 C at mass loading values of 1.3 mg_S_ cm^−2^ (**a**) and 2.5 mg_S_ cm^−2^ (**b**) obtained for WKC-NW/S and WKC-W/S composites.

Finally, a comparison of the long-term cycling stability obtained for both composites at 0.1 C over 200 cycles at two different mass loading values, 1.3 mg_S_ cm^−2^ and 2.5 mg_S_ cm^−2^, is shown in Figure 11. Once again, these results confirm what was observed in Figure 8 and Figure 9. At a mass loading value of 1.3 mg_S_ cm^−2^, the capacity values for the first and second cycles were 875/801 and 1298/1177 mAh g^−1^ sulfur (52/48 and 77/70% of total theoretical capacity) for WKC-NW/S and WKC-W/S, respectively. Whereas after 200 cycles, the corresponding capacity values were 438 and 586 mAh g^−1^ sulfur with a Coulombic efficiency of 99% for both cells. For the high mass loading, WKC-NW/S drastically worsened its electrochemical performance, merely achieving capacity values less than 100 mAh g^−1^ over 100 cycles. Conversely, the washed, non-activated sample, showed a lower initial capacity, but the capacity retention was actually higher. If we compare the capacity values for the 10th and the 200th cycles, a capacity retention of 72% was achieved for the cell with an areal loading of 2.5 mg_S_ cm^−2^, compared to merely 50% capacity retention for the cell with half that areal loading. Thus, from a practical perspective, where capacity retention is considered key, a higher mass loading did not negatively affect the performance, but actually improved it.

Following material characterization, the better electrochemical performance of the washed, non-activated carbons is attributed to both chemical and morphological changes. In turn, the presence of N and O heteroatoms has long been reported to improve polysulfide retention in the carbonaceous matrix, providing active sites for polysulfide anchoring [44,56,57], and it was determined that the washed sample contains a larger O content, as compared to the non-washed sample. The higher S_BET_, pore volume, microporosity, *I_D_*/*I_G_* and diffusion coefficients of WKC-W compared to WKC-NW would also improve the performance [58]. Our hypothesis is that, in this case, both elemental sulfur and the solid discharge products can accommodate better during cycling and the contact between electrolyte and sulfur is facilitated.

To sum up and make a comparison with the literature that use carbons derived from micro-organisms and non-activated carbons as sulfur hosts in Li-S batteries, the more relevant properties from these reports are presented in Table 3. There are very few cathode materials which use carbonized biomass obtained from micro-organisms in their composition and waste materials that are only carbonized, i.e., without using activation processes. Some of them use the micro-organisms as initiators of biological fermentation mixed with waste biomass, avoiding chemical activators or directly used as cathode material. Besides, it should be noted that some of these materials were naturally richer in N, resulting in carbons with natural nitrogen doping, which are known to stabilize the structure of electrodes and restrict the dissolution of polysulfides [21,59]. A hydrophilic/amphiphilic design with excellent trapping capability is provided for the instance of rice husks by the natural carbon sources, cellulose, hemicellulose, and lignin with SiO_2_ particles inserted in the carbon framework [26]. Carbonaceous materials’ hierarchical structure of linked pores, including micro, meso, and macroporous, is a crucial factor that can improve capacity retention and lessen the shuttle effect [24,25,27,58]. The synthetized carbon materials had relatively high sulfur contents. With regard to S_BET_, more variation has been found among the listed carbons, it can be as low as 100 m^2^ g^−1^ or as high as 700 m^2^ g^−1^ for non-activated carbon or 500 m^2^ g^−1^ for a carbon source activated with H_3_PO_4_. The table shows that the electrochemical performance of our Li-S cathodes is quite similar to the listed references, both in the achieved discharge capacity and the relatively mild discharge rates tested.

## 4. Conclusions

A simple synthesis procedure was developed to prepare a novel microbial biomass-derived carbon used as a sulfur host in Li-S batteries. Water kefir grains can be reproduced easily from a sugary and mineralized beverage, and the porous sample is obtained easily after filtration, drying and pyrolysis. Therefore, this protocol is simple, low cost, scalable, environmentally friendly, and comes from a natural and abundant source. Moreover, the fact of not needing a chemical activation step makes it a greener and more sustainable process. WKC-W depicted a specific surface area of 630 m^2^ g^−1^ with a high percentage of micropores which could host a high sulfur content of 68%; the high intensity ratio (*I_D_*/*I_G_*), diffusion coefficients and O and N doping could also improve the performance. When tested as cathode material in Li-S batteries at 0.1 C, the cell achieved an initial capacity of 1298/1019 mAh g^−1^ sulfur and 587/497 mAh g^−1^ sulfur after 200 cycles at high mass loading values of 1.3 and 2.5 mg_S_ cm^−2^, respectively. This work provides an opportunity of converting a biomass-derived carbon into a sulfur host precursor to be used as a cathode in Li-S batteries with promising performance. In order to achieve fully optimized battery cycling, further fine-tuning of the other battery variables is necessary (i.e., anode passivation, electrolyte/sulfur ratio, composition and concentration of the electrolyte, etc.). However, this work represents a first step in the search of microbial biomass-derived carbons to prepare a cathode material for Li-S battery.

## Figures and Tables

**Table 1:** Textural properties for WKC-NW and WKC-W (SBET: specific surface area, VTP: total pore volume, VµP: micropore volume).

Sample	S_BET_ (m^2^ g^−1^)	V_µP_ (cm^3^ g^−1^)	V_TP_ (cm^3^ g^−1^)
WKC-NW	270	0.10	0.11
WKC-W	630	0.23	0.27

**Table 2:** Li^+^ diffusion coefficients D (cm^2^ s^−1^) obtained for the discharge and charge processes for WKC-NW/S and WKC-W/S cells.

	Li^+^ Diffusion Coefficient D (cm^2^ s^−1^)	
	WKC-NW/S	WKC-W/S
Peak A	5.30 × 10^−9^	2.20 × 10^−8^
Peak C_1_	1.53 × 10^−9^	7.50 × 10^−9^
Peak C_2_	1.51 × 10^−8^	3.96 × 10^−8^

**Table 3:** Comparison of carbon materials derived from micro-organisms for Li-S batteries found in the literature.

Sulfur Host	S_BET_ (m^2^ g^−1^)	Sulfur Content (%)	Mass Loading (mg_S_ cm^2^)	Discharge Capacity (mAh g^−1^)	Current Density/Cycle Number	Ref.
Banana peels with yeast	112	74	1.3	700	0.1 C/100	[21]
Green algae	101	63	3.5	757	0.1 C/100	[23]
Fungus *	509	52	1.5–1.6	663	0.1 C/100	[22]
Microalgaes	671	65	1.7–2.0	1031	0.06 C/100	[60]
Yeast	721	65	Not specified	725	0.1 C/400	[59]
Rice husk	525	56	1.0	600	0.1 C/500	[26]
Wood microfiber	586	70	1.3	859	0.2 C/450	[25]
Bark of plane trees	528	48	3.2–4.2	608	0.1 C/60	[27]
Filter paper	581	Not specified	1.9	500	0.2 C/100	[24]
Water kefir carbon	630	68	1.3 2.5	743 611	0.1 C/100 0.1 C/100	This work

* Activated carbons.

## Data Availability

Not applicable.

## Supplementary Materials

The following supporting information can be downloaded at: https://www.mdpi.com/article/10.3390/ma15248856/s1.
