# Supplementary material for: Water Kefir Grains—Microbial Biomass Source for Carbonaceous Materials Used as Sulfur-Host Cathode in Li-S Batteries
Source: Materials (Basel). 2022 Dec 12;15(24):8856. doi: 10.3390/ma15248856 (PMC9785453; doi:10.3390/ma15248856)
Supplement: Supplementary file 1 [file materials-15-08856-s001.zip › materials-2044445-supplementary.pdf]

Supporting Information

Water Kefir Grains - Microbial Biomass Source for Carbonaceous Materials Used as Sulfur-host Cathode in Li-S Batteries

Ana L. Páez Jerez[1], M. Fernanda Mori[2], Victoria Flexer[1] and Alvaro Y. Tesio[1*]

[1] CIDMEJu (CONICET-Universidad Nacional de Jujuy), Centro de Desarrollo Tecnológico General Savio, Av. Martijena S/N, Palpalá, Jujuy, 4612, Argentina

[2] Instituto de Investigaciones en Catálisis y Petroquímica, CONICET, Facultad de Ingeniería Química, Universidad Nacional del Litoral, Santiago del Estero 2829, S3000AOM Santa Fe, Argentina

* Correspondence: atesio@cidmeju.unju.edu.ar

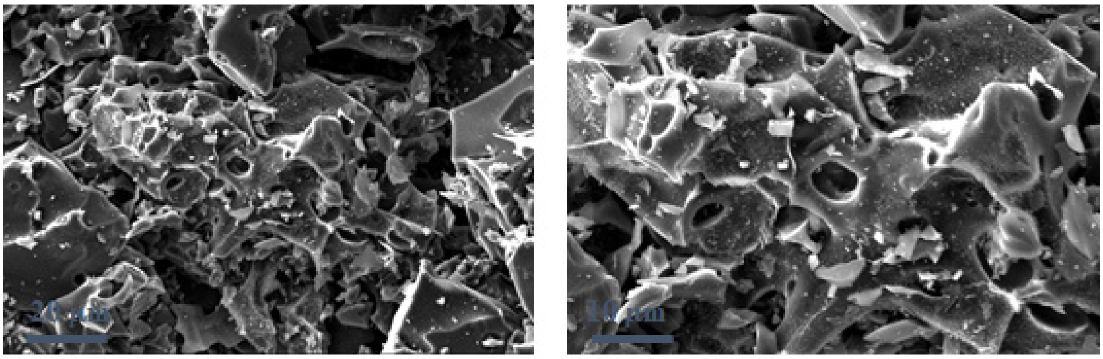

Figure S1. SEM images obtained for washed water kefir carbons measured at 20 and 10 μm.

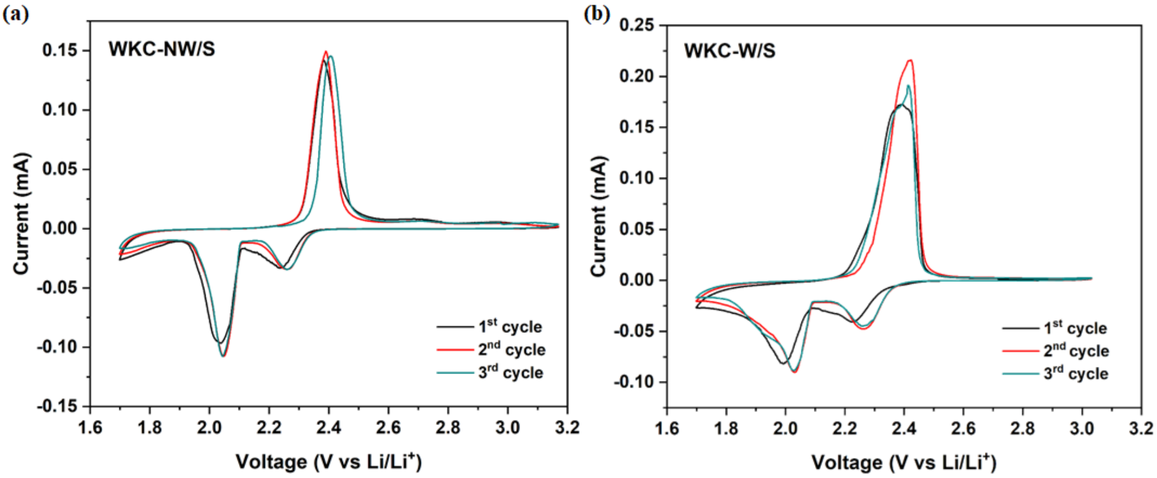

Figure S2. Three consecutive CV curves for WKC-NW/S (a) and WKC-W/S (b).

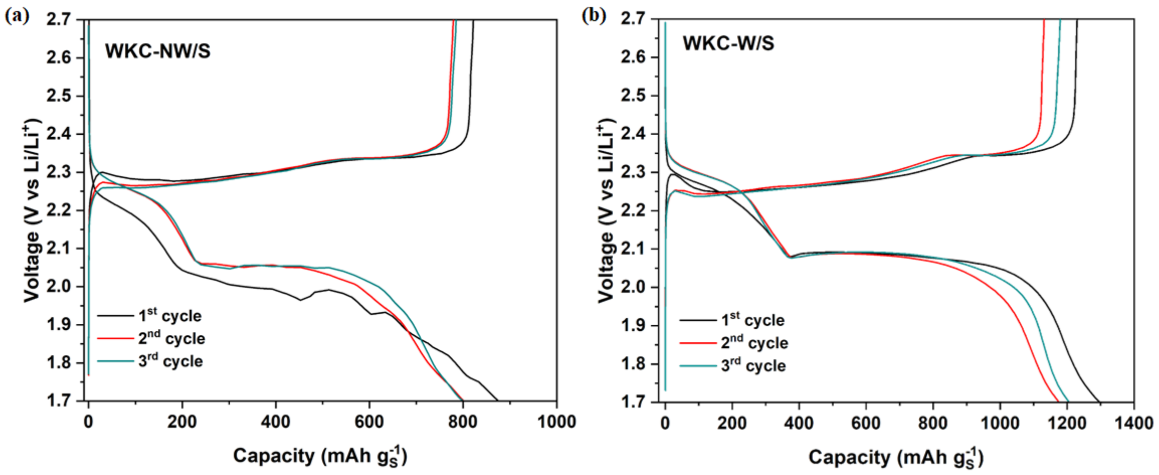

Figure S3. Three consecutive GCD profiles for WKC-NW/S (a) and WKC-W/S (b) at 0.1 C.

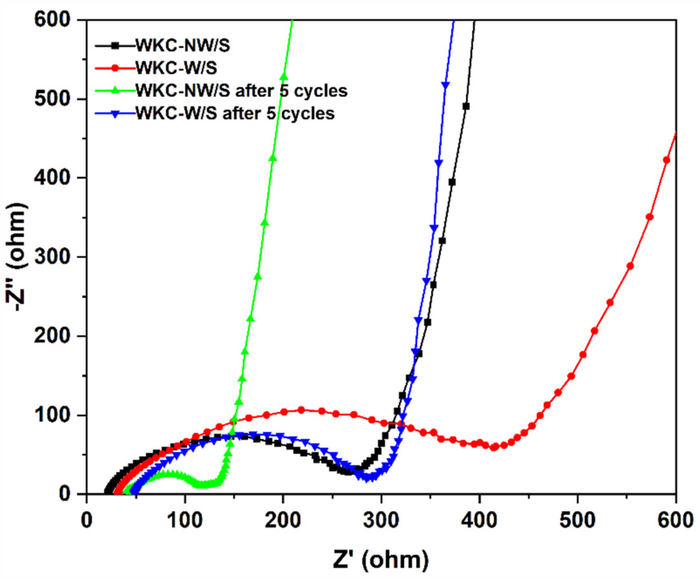

Figure S4. EIS spectra for WKC-NW/S and WKC-W/S before and after 5 cycles at 0.1 C.
